# Traditional Chinese medicine for acute myelocytic leukemia therapy: exploiting epigenetic targets

**DOI:** 10.3389/fphar.2024.1388903

**Published:** 2024-06-04

**Authors:** Xinlong Gao, Xu Zuo, Tianjiao Min, Yu Wan, Ying He, Beier Jiang

**Affiliations:** ^1^ Naval Medical Center of PLA, Shanghai, China; ^2^ College of Food Science and Technology, Shanghai Ocean University, Shanghai, China

**Keywords:** acute myeloid leukemia, hematological malignancy, epigenetics, traditional Chinese medicine, bioactive compounds

## Abstract

Acute myeloid leukemia (AML) is a heterogeneous hematological malignancy with historically high mortality rates. The treatment strategies for AML is still internationally based on anthracyclines and cytarabine, which remained unchanged for decades. With the rapid advance on sequencing technology, molecular targets of leukemogenesis and disease progression related to epigenetics are constantly being discovered, which are important for the prognosis and treatment of AML. Traditional Chinese medicine (TCM) is characterized by novel pharmacological mechanisms, low toxicity and limited side effects. Several biologically active ingredients of TCM are effective against AML. This review focuses on bioactive compounds in TCM targeting epigenetic mechanisms to address the complexities and heterogeneity of AML.

## 1 Introduction

Acute myeloid leukemia (AML), the most prevalent acute leukemia in adults, arises from aberrant transformation of primitive hematopoietic stem cells (HSCs) and progenitor cells (HSPCs), resulting in abnormal proliferation and impaired differentiation of immature bone marrow progenitors (Khwaja et al., 2016). Leukemia cells possess the capacity to infiltrate bone marrow, blood, and other tissues, leading to rapid progression of clinical manifestations (Döhner et al., 2015). Multiple studies have demonstrated that hematopoietic stem cells (HSCs) or progenitor cells can transform into leukemia stem cells (LSCs), which possess the ability of self-renewal and sustained proliferation, leading to the development, progression, and multidrug resistance of AML (Stelmach and Trumpp, 2023). Mutations in specific genes, such as FLT3, NPM1, CEBPA, RUNX1, etc., also contribute to an increased number of LSC (Papaemmanuil et al., 2016). These mutations may be present in early clones during the initial stages of the disease, leading to alterations in gene expression within signaling pathways involved in hematopoietic cell proliferation and differentiation, leukemia development and maintenance of LSCs, thereby impacting the occurrence, progression, and prognosis of AML (Egan and Schimmer, 2023). Environmental factors, including prolonged exposure to specific chemicals (e.g., chlorobenzene, cyclophosphamide) or radioactive substances, can also elevate the risk of AML (Bueso-Ramos et al., 2015). Furthermore, abnormal epigenetic regulation is commonly observed in patients with AML. This aberration can result in the abnormal suppression or overexpression of crucial genes, thereby impacting the proliferation, apoptosis, and differentiation of leukemia cells. Consequently, epigenetic modification is now recognized as a pivotal factor driving the initiation and progression of AML (Nuno et al., 2023).

In the past few decades, AML has typically been managed with intensive chemotherapy utilizing anthracyclines or anthranones in combination with cytarabine (known as a “3 + 7″regimen), followed by multiple courses of consolidation chemotherapy or hematopoietic stem cell transplantation (Döhner et al., 2017). However, the long-term survival rate after standard chemotherapy is only approximately 20%–40%, and most AML patients are at risk of relapse, with some failing to achieve complete remission (Zappasodi et al., 2018). In recent years, the emergence of demethylating agents such as decitabine (DAC) and azacytidine (AZA) has provided novel therapeutic options for AML patients who are not suitable candidates for standard chemotherapy regimens. These agents modulate DNA methylation patterns and reactivate silenced tumor suppressor genes, thereby promoting cancer cell differentiation and apoptosis (Xu and Yu, 2020). With the advancement of high-throughput sequencing technology, our comprehension of AML epigenetics is deepening, thereby positioning epigenetic modification as a potential novel target for future diagnosis and treatment strategies in AML. However, further investigation is warranted to gain comprehensive insights into the mechanisms underlying the efficacy of epigenetic therapies, address concerns regarding drug resistance, and explore optimal combinations with traditional treatments to enhance treatment outcomes for patients with AML.

Traditional Chinese medicine (TCM) is extensively utilized for the prevention and treatment of a wide range of diseases. In recent years, an increasing number of studies have emphasized the significance of TCM in cancer therapy. Compared with conventional therapies, TCM has the advantage of multiple components and targets with limited side effects, making it particularly suitable for treating complex diseases, especially cancer (Wang et al., 2021). After years of basic and clinical research, TCM has made significant achievements in the field of tumor treatment. Various anti-tumor drugs successfully extracted and developed from Chinese herbal medicine, thereby providing a rich material foundation for the advancement of novel anti-cancer drugs (Luo et al., 2019). Currently, TCM has emerged as a pivotal therapeutic strategy for leukemia (Huang et al., 2017), effectively extending the survival and enhancing the quality of life of patients (Chen and Zhuang, 2024). Vincristine, a dimer-indo-alkaloid extracted from the leaves of Gatharanthus roseus, is effective to treat acute lymphocytic cell leukemia (ALL) clinically (Cragg and Newman, 2009). Arsenic trioxide (ATO), the most widely used arsenical as an anti-cancer drug, has been clinically approved in the treatment of APL targeting the PML/RARα oncofusion protein (Nayak et al., 2010). Notably, TCM exhibits distinctive attributes including limited toxicities, wide accessibility at a cost-effective manner, and suitability for long-term administration. Therefore, the use of TCM in combination with chemotherapy and bone marrow transplantation for leukemia treatment has gained increasing recognition with special clinical benefits. Despite the extensive research into leukemia therapy of TCM for decades, most TCM prescriptions still lack clear targets and signaling pathways (Zhu et al., 2022). This article comprehensively reviews the AML and the significance of epigenetics in its pathogenesis, as well as the current status of TCM in improving AML therapeutic effects.

## 2 Epigenetics

**FIGURE 1:**
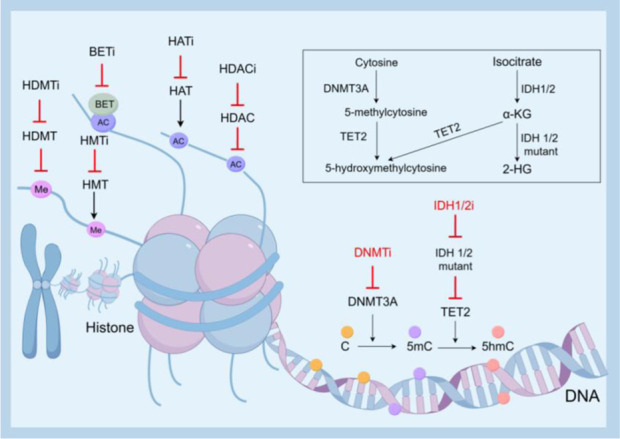
Epigenetic pathogenesis of AML and mechanisms of targeted therapy.

In recent years, the crucial role of epigenetic modification in the processes of tumorigenesis, proliferation, and metastasis has gained widespread recognition in recent years. These modifications encompass alterations in DNA methylation, histone modification, and non-coding RNA rather than changes in DNA sequence itself. In hematologic tumors such as AML, epigenetic dysregulation can lead to tumor cell proliferation and metastasis, affect the expression of key genes, and is an important target for AML treatment. In hematologic tumors such as AML, epigenetic dysregulation can lead to tumor cell proliferation and metastasis, affect the expression of key genes, and is an important target for AML treatment. The common epigenetic pathogenesis of AML and mechanisms of targeted therapy are shown in Figure 1.

### 2.1 DNA methylation in AML

DNA methylation is a pivotal epigenetic modification mechanism that catalyzes the addition of a methyl group to the five carbon atoms of the cytosine molecule, resulting in the formation of 5-methylcytosine, which plays an indispensable role in various fundamental cellular processes (Jaenisch, 1997; Schaefer et al., 2007). Initially, the investigation of abnormal Global hypomethylation or hypermethylation of the promoter region is often observed in AML cells. The induction of gene silencing can be attributed to highly methylated promoter regions, whereas the promotion of gene expression may arise from poorly methylated CpG off-island regions (Baylin and Jones, 2011). The aberrant methylation patterns in adult AML primarily result from mutations affecting the DNMT3A, TET2, and IDH1/2 enzymes.

DNMT1, DNMT3A, and DNMT3B in the DNMT family are methyltransferases that catalyze DNA methylation and are responsible for adding methyl groups to cytosine residues of CpG dinucleotides to maintain the methylated state of HSCs and progenitors (Li et al., 2013; Liu X. et al., 2015). DNMT1 is primarily responsible for maintaining the DNA methylation state, whereas DNMT3A and DNMT3B facilitate *de novo* DNA methylation (Cui and Xu, 2018). DNMT3A mutations can result in dysregulation of DNA methylation, thereby eliciting global alterations in gene expression patterns in hematological malignancies. This perturbation typically enhances the self-renewal capacity of blood cells while compromising their normal differentiation process (Mizuno et al., 2001; Li et al., 2013; Deveau et al., 2015). Approximately 12–22 percent of patients with AML harbor a heterozygous DNMT3A gene mutation (Sun et al., 2018). The most prevalent mutation occurs at the arginine (R882) site and is associated with heightened proliferation of HSCs as well as resistance to anthracycline chemotherapy (Ohgami and Arber, 2015). In AML, restoration of normal molecular function can be achieved through hypomethylation-mediated inhibition of DNMT. Demethylation drugs, also known as DNMT inhibitors or HMAs, such as DAC and AZA, have been approved for clinical treatment of hematological malignancies and the overwhelming majority of studies demonstrate encouraging outcomes (Leisch et al., 2022).

TET2 is a prevalent epigenetic modifier in hematological malignancies. Functioning as a dioxygenase that utilizes α-ketoglutaric acid (α-KG), Fe^2+^, and O_2_, TET2 catalyzes the hydroxylation of DNA methylcytosine (5 mC), leading to its conversion into 5-hydroxymethylcytosine and subsequent generation of other derivatives, ultimately facilitating demethylation for DNA formation. TET2 mutations can induce a pre-leukemic state in HSCs, while preserving their capacity to differentiate into fully mature blood cells. However, upon acquisition of additional genetic alterations, these preleukemic stem cells can undergo transformation into leukemic initiation cells and drive the progression towards full-blown leukemia (Chan and Majeti, 2013; Corces-Zimmerman and Majeti, 2014; Sato et al., 2016).

IDH mutations are detected in approximately 20% of patients with AML (DiNardo et al., 2015). IDH catalyzes the oxidative decarboxylation of isocitric acid to α-KG. IDH mutations convert α-KG into the carcinogenic metabolite 2-hydroxy-glutaric acid (2-HG), which accumulates in tumor cells, leading to aberrant DNA or histone hypermethylation. Moreover, the interaction between IDH1/2 and TET2 exerts an impact on the differentiation of hematopoietic cells, thereby driving leukemogenesis (Yen et al., 2017; Zeidner, 2020). In preclinical models of AML, targeted inhibition of IDH mutations effectively attenuates intracellular 2-HG levels and reverses the progression of AML (Sulkowski et al., 2017). It has been observed that mutations in TET2 and IDH1/2 exhibit mutual exclusivity among AML patients. Given that TET2 facilitates DNA demethylation via the α-ketoglutarate-dependent pathway, the presence of 2-hydroxyglutaric acid resulting from IDH1/2 protein mutation can competitively inhibit TET2 function (Xu et al., 2011). Therefore, IDH1/2 mutations facilitate the progression of leukemia by impeding myeloid differentiation, disrupting TET2-mediated DNA demethylation, and aberrantly augmenting c-Kit expression (Figueroa et al., 2010).

### 2.2 Histone modifications

Histone modifications including methylation, acetylation, phosphorylation, adenylation, ubiquitination and ADP ribosylation (Jezek and Green, 2019), play a pivotal role in the regulation of chromatin structure remodeling and genome expression, thereby exerting significant influence on the pathogenesis and progression of numerous diseases (Suganuma and Workman, 2011). The enzymes involved in histone modification encompass histone acetyltransferase (HAT), histone deacetylated transferase (HDAC), histone methyltransferase (HMT), and histone demethylase (HDMT). Among them, HDAC and HDMT collaboratively facilitate the removal of histone modifications (Müller et al., 2013).

Histone acetylation is a reversible process that occurs on histone tail residues, regulated by histone HATs and deacetylases HDACs (Vogelauer et al., 2000), which play a crucial role in gene transcription regulation. Recent studies have revealed that abnormal function of histone acetylation is strongly linked to leukemia occurrence, with numerous chromosome alterations involving HDAC protein and its dysregulated activity (Eberharter and Becker, 2002), resulting in perturbation or overexpression of key factors leading to leukemia onset. Additionally, elevated levels of HDACs significantly downregulate tumor suppressor genes while promoting tumor angiogenesis and cell migration ultimately leading to proliferation, invasion, and metastasis in cancer cells. In AML specifically, increased HDAC expression disrupts cell cycle regulation through aberrant signaling pathways affecting cellular proliferation, differentiation, and apoptosis. Conversely reducing HDAC expression enhances activity of specific tumor suppressor genes facilitating their transcriptional activation thereby suppressing AML cell proliferation while inducing apoptosis (Barneda-Zahonero and Parra, 2012; Beyer et al., 2019).

The methylation modification of histones occurs on lysine and arginine residues, which is co-regulated by HMT and HDMT. Aberrant patterns of histone methylation can result in abnormal proliferation and survival of AML stem cells, thereby promoting the development of AML. EZH2, a histone lysine methyltransferase (HKMT), catalyzes the PRC2 protein complex and is frequently mutated or overexpressed in myeloid malignancies (Cao et al., 2002). Mutated or overexpressed EZH2 can inhibit the differentiation of AML stem cells, thus promoting both the occurrence and progression of AML (Chen and Zhuang, 2024). The MLL gene on chromosome 11q23 encodes an H3K4 methyltransferase, which plays a role in histone modification and influences HOX gene expression and Wnt signaling (Milne et al., 2002). MLL mutations occur in 5%–7% of newly diagnosed AML cases and are linked to unfavorable outcomes. Interactions between MLL fusion proteins and DOT1L have been demonstrated to drive the progression of leukemia (Bernt et al., 2011).

### 2.3 Non-coding RNAs in AML pathogenesis

In recent years, with the rapid advancement of functional genomics and high-throughput sequencing technology, researchers have discovered that a diverse range of non-coding RNAs (such as lncRNA, miRNA, etc.) play a pivotal regulatory role in the initiation and progression of hematological malignancies (Dahariya et al., 2019). miRNAs, short non-coding RNAs that bind to target gene mRNA to regulate their expression, often exhibit abnormal expression patterns in AML patients. Some miRNAs are overexpressed or silenced, thereby impacting key biological characteristics of leukemia cells such as proliferation, apoptosis, and differentiation. Furthermore, aberrant expression of certain lncRNAs also influences the proliferation capacity, survival ability, invasion potential of leukemia cells while promoting the occurrence and progression of AML. Due to their significant involvement in AML pathogenesis, ncRNAs are considered promising therapeutic targets.

Additionally, aberrant expression of non-coding RNA is closely associated with drug resistance in patients with AML. For example, HOTAIR expression levels were significantly upregulated in patients with refractory and relapsing AML and in AML resistant cell lines (K562/A02). Inhibition of HOTAIR expression inhibited cell proliferation and enhanced the sensitivity of K562/A02 cells to adriamycin (Li ML. et al., 2020). In addition, miRNAs can regulate the proliferation and apoptosis of AML cells. The expression of miR-182-5p was significantly upregulated in AML cells, and inhibition of miR-182-5p activity could significantly reduce the proliferation of AML cells and reverse cisplatin resistance (Zhang et al., 2018). Further investigation and elucidation into the specific mechanisms underlying various ncRNAs will provide novel insights and directions for comprehending the etiology and treatment of AML.

## 3 Bioactive compounds in TCM targeting epigenetic mechanisms in the treatment of AML

### 3.1 Clinical applications

Significant progress has been achieved in the treatment of tumors by combining traditional TCM with Western medicine (WM), following years of extensive basic and clinical research. WM primarily employs chemotherapy, bone marrow transplantation, gene therapy, and immunotherapy for leukemia treatment (Liu, 2021), while TCM typically employs the methods of clearing heat and detoxifying, strengthening body resistance and promoting blood circulation and removing blood stasis according to the fundamental principles of TCM (Ma et al., 2021). The bioactive compounds of TCM serve as the fundamental basis for its pharmacological efficacy, exhibiting commendable clinical performance in the AML treatment. Arsenic trioxide (ATO) is one of the most common drugs used in the frontline treatment of APL that act through targeting and destabilizing the PML/RARα oncofusion protein (Yu et al., 2023). ATO together with all-trans retinoic acid (ATRA) lead to durable remission of more than 90% non-high-risk APL patients, turning APL treatment into a paradigm of oncoprotein targeted cure (Langdon et al., 2024). The plant-derived agent homoharringtonine (HHT), derived from the Chinese tree Cephalotaxus harringtonia var. drupacea, exhibits significant anti-leukemia effects and demonstrates efficacy in the treatment of acute and chronic myeloid leukemia, as well as myelodysplastic syndrome. HHT has been approved by the US FDA for treating CML. In China, HHT is extensively utilized in the management of AML, and “HAA” program (homoharringtonine + cytarabine + aclarubicin) has become the first-line treatment for low- and medium-risk AML patients in China through multicenter phase III clinical trials currently.

### 3.2 Epigenetic mechanisms

Given the critical role of epigenetic aberrations in AML pathogenesis, epigenetic therapies have emerged as promising strategies. Drugs targeting DNA methylation (e.g., azacitidine and decitabine) or HDAC inhibitors have shown clinical benefits, underscoring the potential of targeting the epigenome for AML treatment. Many TCM and their active ingredients, such as curcumin, resveratrol, and epigallocatechin-3-gallate (EGCG), have been shown to possess epigenetic modulating activities. They can alter the methylation patterns of specific genes or influence histone modifications, potentially reversing the malignant phenotype of AML cells. Epigenetic modulation by TCM compounds may possess the potential to overcome drug resistance observed in standard therapies for AML, a significant challenge in the treatment of this disease. Furthermore, the modulation of epigenetic markers by certain TCM compounds can enhance the sensitivity of AML cells to conventional chemotherapeutic agents, thereby potentially reducing the required chemotherapy dosage and minimizing associated side effects. Several mechanisms have been elucidated that explain for the modulatory effects of TCM compounds on epigenetic targets as depicted in Tables 1– 3. Here’s a comprehensive look of the underlying mechanism on epigenetic targets.

**TABLE 1:** Screening of DNA methylation-related targets in the treatment of acute myeloid leukemia with traditional Chinese medicine and study on the mechanism of action of active components.

TCM ingredient	Category	Mechanism	Research phase	Refers
Arsenic trioxide	Inorganic substance	Restore the hypermethylation of the TMS1 gens; Induce DNA hypomethylation and inhibit DNMT	Clinical research	(Di Croce et al., 2002; Khaleghian et al., 2014; Li et al., 2015)
Curcumin	Polyphenols	Inhibit DNA methylation	Preclinical studies	Yu et al. (2013)
Dimethoxycurcumin	Curcumin derivatives	Induce methylation of promoter gene expression	Preclinical studies	Hassan et al. (2015)
Oridonin	Tetracyclic diterpenoids	Inhibit DNMT3A^R882^ mutation-driven AML	Preclinical studies	Liao et al. (2021)
Homoharringtonine	Alkaloid	Reduced overall DNA 5-hydroxymethylcytosine abundance	Clinical research	Li et al. (2020b)
Quercetin	Flavonoid	Reduce DNMT1 and DNMT3A; Induce demethylation of DAPK1 genes	Preclinical studies	Alvarez et al. (2018)
Epigallocatechin gallate	Tea polyphenols	Reduce hypermethylation of CHD5 gene; Inhibit DNMT1	Preclinical studies	Wu et al. (2020)

**TABLE 2:** Screening of histone modification related targets and study on the mechanism of action of effective components in the treatment of acute myeloid leukemia with Chinese medicine.

TCM ingredient	Category	Mechanism	Research phase	Refers
Dimethoxycurcumin	Curcumin derivatives	Increase the H3K36me3 marker near the hypermethylated gene promoter region	Preclinical studies	Hassan et al. (2015)
Quercetin	Flavonoid	Induce demethylation of DAPK1 genes and acetylation; Activate Promote histone H3 acetylation	Preclinical studies	(Alvarez et al., 2018; Lee et al., 2011)
Epigallocatechin gallate	Tea polyphenols	Inhibit HDAC	Preclinical studies	Moradzadeh et al. (2018)
Acanthopanax senticosus	Flavonoid	Inhibit HADC	Preclinical studies	Wang et al. (2016a)
ginsenoside	Saponins	Inhibit HDAC1, HDAC2 and HDAC6; Enhance histone H3 acetylation and HAT activity	Preclinical studies	Liu et al. (2015b)

**TABLE 3:** Screening of non-coding RNA-related targets and study on the mechanism of action of active components in the treatment of acute myeloid leukemia with traditional Chinese medicine.

TCM ingredient	Category	Mechanism	Research phase	Refers
Curcumin	Polyphenols	Inhibit miRNA-20a-5p	Preclinical studies	Liu et al. (2021)
Ginsenoside	Saponins	Targeting the miR-3677-5p/CXCL12 axis	Preclinical studies	Ma et al. (2022)
Tanshinone IIA	Terpene	Modulate the MAPK/ERK1/2 pathway by miR-497	Preclinical studies	Nie et al. (2020)
Resveratrol	Flavonoid	Regulates miR-17, miR-92b, miR-181a, and miR-222	Preclinical studies	Iravani Saadi et al. (2023)
Gallic Acid	Polyhydroxy fatty acids	Similar to Resveratrol	Preclinical studies	Iravani Saadi et al. (2023)
Piperine	Alkaloid	Similar to Resveratrol	Preclinical studies	Iravani Saadi et al. (2023)
Matrine	Alkaloid	Targeting miR-495-3p and miR-543	Preclinical studies	Lei et al. (2024)

#### 3.2.1 DNA methylation

The aberrant DNA methylation patterns, specifically hypermethylation of tumor suppressor genes and hypomethylation of oncogenes, have been observed in AML. The altered methylation states can impede normal hematopoietic differentiation, thereby contributing to the perpetuation of leukemic conditions. The studies on TCM regulating DNA methylation modification primarily focus on the entire genome and abnormal methylation status through active ingredients. The mechanism underlying this process mainly involves the regulation of DNMTs expression or related signaling pathways.

ATO is widely utilized in the frontline treatment of APL, which involves destabilization of the PML/RARα fusion protein. A previous research demonstrated that PML/RARα directly interacted with DNMTs, including DNMT1 and DNMT3A, resulting in hypermethylation of downstream genes to promote leukemogenesis (Di Croce et al., 2002). Khaleghian A et al. (Khaleghian et al., 2014) proved that ATO directly inhibited the mRNA expression of DNMTs (DNMT1, DNMT3A, and DNMT3B) in the APL cell line NB4. Similarly, As_2_O_3_ could inhibit DNMTs and restore the hypermethylation status of the TMS1 gene, thereby inducing apoptosis in K562 cells via downregulation of Bcl-2/Bax expression (Li et al., 2015). Furthermore, a recent research showed the suppression of DNMTs expression by ATO induced DNA hypomethylation at the promoter region of cell cycle related genes which could potentially elucidate the mechanism behind ATO-induced cell cycle arrest in NB4 cells s and its efficacy in eradicating APL (Hassani et al., 2018). The collective findings indicated that ATO may possess the capacity to reverse aberrant DNA methylation patterns, thereby contributing to the treatment of leukemia. Curcumin in TCM is primarily aimed at enhancing blood circulation and resolving blood stasis, as it has been extensively investigated for its remarkable anti-tumor and antioxidant activity. Curcumin inhibited DNA methylation by covalently blocking the catalytic thiolate of DNMT1 and reducing the expression of tumor suppressor gene Sp1 Transcription Factor (Sp1) and Transcription factor p65 (p65) in AML cell lines *in vitro* and *in vivo* (Yu et al., 2013). Dimethoxycurcumin (DMC), the synthetic structural analogue of curcumin, could induce the expression of genes with methylated promoters without reversing DNA methylation and increased the H3K36me3 mark near the promoter region of hypermethylated genes (Hassan et al., 2015). The combination of DMC and DNMT inhititors decitabine could enhance gene re-expression of promoter-methylated genes and significantly increased H3K27 acetylation (Hassan et al., 2016). Min Liao et al. revealed that the occurrence of DNMT3A mutations in *de novo* AML patients was approximately 20%, with more than 50% of these mutations being heterozygous missense alterations within the methyltransferase domain at residue R882 (Liao et al., 2021). The presence of DNMT3A R882 mutations in AML patients confered resistance to anthracycline chemotherapy and drives relapse. Oridonin, an ent-kaurene diterpenoid isolated from the Chinese herb Rabdosia rubescens, effectively suppressed DNMT3A R882 mutation-driven AML through the induction of apoptosis and necroptosis. Currently, the TCM Donglingcao, which is mainly composed of Oridonin, is being studied in clinical research for its potential in treating DNMT3A-mutated AML. The alkaloid Homoharringtonine (HHT), isolated from the cephalotaxus hainanensis, has been approved by FDA for the treatment of chronic myeloid leukemia (CML). Moreover, HHT is extensively utilized in China for the management of AML. The FLT3 gene is a crucial downstream target of the HHT/SP1/TET1/5hmC signaling pathway. Treatment with HHT significantly reduced global DNA 5-hydroxymethylcytosine abundance by targeting the SP1/TET1 axis (Li C. et al., 2020). Quercetin, a naturally occurring flavonoid, has demonstrated remarkable potential as a multipotent bioflavonoid for the prevention and treatment of cancer. The treatment with Quercetin significantly decreased the expression of DNMT1 and DNMT3a and induced demethylation of the pro-apoptotic BCL2L11, DAPK1 genes, which further promoted cell death in leukemia (Alvarez et al., 2018). Quercetin also induced Fas ligand-related apoptosis through activating the ERK and JNK pathways and promotion of histone H3 acetylation in leukemia cells (Lee et al., 2011). EGCG, the main active catechin in green tea, is involved in numerous biological mechanisms related with cancer development and progression as a potential anti-cancer agent. EGCG dose-dependently reduced hypermethylation of CHD5 gene by downregulating the expression of DNMT1 in KG-1 and THP-1 cells, which promoted AML cell apoptosis (Wu et al., 2020). Besides, EGCG effectively suppresses the proliferation and cell cycle progression of NB4 cells by inhibiting the expression of DNMT1 and DNMT3a, thereby inducing a downregulation in the methylation of DAPK1 gene (Shi et al., 2018).

#### 3.2.2 Histone modifications

Histone modification is a crucial mechanism in the regulation of epigenetics. By modifying histones through acetylation and methylation, it is possible to regulate the accessibility of chromosomes and genes. Abnormal histone modifications are frequently observed in AML patients, which can result in aberrant expression of leukemia-related genes including oncogene overexpression and tumor suppressor gene silencing. Specific histone modifications may serve as potential targets for AML treatment. Targeted intervention aimed at correcting abnormal histone modifications in leukemia cells can be designed to regulate cell proliferation and survival, ultimately improving therapeutic outcomes.

Compared with DNA methylation, histone modifications in APL pathogenesis and treatment response may have been even more significant. Ji Li et al. showed that ATO induced apoptosis by promoting histone H3 phosphoacetylation of Caspase-10 in NB4 cells (Li et al., 2002). Another study also demonstrated that arsenic exposure significantly promoted global histone acetylation (Ramirez et al., 2008). Besides, ATO nanoparticles activated p21(WAF1/CIP1) gene by promoting acetylation of H3K14 and phosphorylation of H3S10, thereby leading to apoptosis in the prostate cancer cells, implaying arsenic might exert apoptotic effects by modulating histone modifications (Jadhav et al., 2016). EGCG was shown to reduce proliferation and cause apoptosis in NB4 cells and it also inhibited HP1α and DNMT1 protein expression and H3K9me3 modification (Vitkeviciene et al., 2018). Administration of EGCG enhanced differentiation of HL-60 cells via inhibition of PML-RARα and HDAC1 (Moradzadeh et al., 2018). Beyond that, another research also proved the inhibitory effect of EGCG on DNMT and HDAC in NB4 and HL-60 cells. It effectively regulated chromatin structure via inhibiting epigenetic modifiers, including DNMT1, HDAC1, and HDAC2. Furthermore, the research demonstrated a downregulation of the G9a gene expression and decreased levels of histone H3K9me2 modification catalyzed by G9a (Borutinskaitė et al., 2018). Suhila Sawesi et al. explored the effect of curcumin and DMC on a range of histone posttranslational modifications and activity. The research findings revealed that Curcumin and DMC effectively inhibited the activity of HKMTs enzymes, which specifically targeted the H3K4, H3K9, and H3K27 marks. Additionally, these two compounds were found to enhance the enzymatic activity of HKDMs enzymes such as LSD1, JARID, and JMJD2 (Sawesi et al., 2022). Acanthopanax senticosus is a multifunctional bioflavonoid with great potential in the prevention and treatment of malignant tumors. Qing-Yuan Wang et al. proved that Acanthopanax senticosus demonstrated a dose- and time-dependent ability to induce apoptosis of human leukemia HL-60 cells, while also exhibiting potential as a HADC inhibitor (Wang QY. et al., 2016). Quercetin was exhibited to enhance the acetylation of H3 and H4 in the promoter regions of the pro-apoptotic BCL2L11, DAPK1 genes, which subsequently promoted cell death in leukemia (Alvarez et al., 2018). The proliferation of KG1-α cells was effectively suppressed by ginsenoside through the downregulation of HDAC1, HDAC2, and HDAC6, accompanied by enhancing histone H3 acetylation and increasing HAT activity (Liu ZH. et al., 2015). The expression of CBP, EP300, and SIRT3 in HL-60/ADR and KG1-α cells was upregulated by berberine, all of which belonged to the class III HDACs, accompanied by a significant reduction in the protein levels of H3K27me3 after treatment (Wang Z. et al., 2016).

#### 3.2.3 RNA-based mechanisms

The expression pattern of ncRNA can serve as a biomarker for AML, playing a crucial regulatory role in the onset and progression of AML, and offering potential applications in disease diagnosis, prognosis assessment, and treatment monitoring. Modulating abnormally expressed miRNAs or lncRNAs can affect the biological behavior of AML cells with potential therapeutic effects on leukemia cells.

The ATO treatment decreased the expression of let-7d and miR-766, thereby significantly suppressing the expression of pro-apoptotic genes caspase-3 and Bax (Liang et al., 2013). Similarly, ATO led to the downregulation of three miRNAs (miR-17, miR-20a, and miR-106a) targeting SIRPα through the activation of β-catenin and c-Myc signaling pathways, thereby significantly contributing to ATO-induced apoptosis in APL cells (Pan et al., 2016). Another study has also confirmed that The ATO treatment modulated numerous cancer-related miRNAs in the APL cell line, most of which were involved in cell cycle arrest and apoptosis (Ghaffari et al., 2012). In conclusion, miRNA serves as a crucial mediator for ATO to exert its anti-cancer activity in APL (Maimaitiyiming et al., 2020). The leukemia cells of M5 patients with EMI showed low miR-3677-5p expression and high CXCL12 and CXCR4 mRNA levels, which could serve as indicators for extramedullary infiltration and poor prognosis. Ginsenoside Rk3, a main active ingredient of ginsenosides, exerted inhibitory effects on proliferation, migration, and invasion in SHI-1 cells by targeting the miR-3677-5p/CXCL12 axis (Ma et al., 2022). Besides, Tanshinone IIA regulated MAPK/ERK1/2 pathway through miR-497 to inhibit cell proliferation in human OCI-AML3 cells (Nie et al., 2020). Resveratrol, Gallic Acid, and Piperine could regulate miR-17, miR-92b, miR-181a, and miR-222 in HL-60 cells and exhibited positive therapeutic effects on AML (Iravani Saadi et al., 2023). Curcumin attenuated Adriamycin-resistance of AML by inhibiting miRNA (miR)-20a-5p, thus suppressing the proliferation and migration and blocking the cell cycle progression of HL-60 cells (Liu et al., 2021). Matrine, a natural alkaloid isolated from the root and stem of the legume plant Sophora, exerts various pharmacological effects through multiple signaling pathways. It was identified as a great anti-tumor agent on AML through the inactivation of the JAK/STAT pathway mediated by the lncRNA LINC01116/miR-592 axis (Zhang et al., 2022). Similarly, Matrine inhibited the progression of AML by selectively targeting miR-495-3p and miR-543, leading to the attenuation of PDK1 expression and subsequent repression of the Wnt/β-catenin signaling pathway (Lei et al., 2024).

## 4 Challenges and perspectives

Although recent research has greatly enhanced our understanding of TCM oncology, significant challenges and limitations remain for its widespread clinical use. First, the standardization of TCM raises a multitude of concerns. TCM consisting of multiple herbs, are characterized by their complexity and diversity. Each herb has its chemical constituents, and the overall effects of these formulas depend on the proportions and quality of the herbs utilized. Furthermore, the quality of herbs may vary due to factors like soil quality, harvest time, processing techniques, and storage conditions. These variations can potentially impact the safety and effectiveness of herbal formulations. Unlike WM, where drugs have a specific dose and purity, many TCMs lack the standardized dosages and preparation methods, which can lead to inconsistencies in efficacy and potential side effects. Without proper standardization, the potential interactions between TCM and WM may also be unpredictable. While numerous studies have looked into the benefits of TCM, there remains a necessity for more rigorous and high-quality randomized controlled trials to establish the safety and efficacy of TCM. Thus there’s a balance to strike between preserving traditional methods and knowledge and integrating modern scientific standards and validation. The regulatory differences and ethical concerns are also need to be addressed. The regulatory framework for TCM, varies from one country to another. In certain jurisdictions, TCM products may be marketed as dietary supplements, while in others they are classified as pharmaceutical drugs. The utilization of certain TCM involves the utilization of ingredients derived from endangered plants or animals. Therefore, any standardization efforts should take into account the sustainable and ethical sourcing of these ingredients. Secondly, TCM-WM interactions occur when a medication and an herb or herbal supplement influence each other’s pharmacokinetics or pharmacodynamics. These interactions can either enhance or decrease the effectiveness of the drug or herb, lead to unexpected side effects, or even produce toxic reactions. Third, despite the efficacy of TCM in tumor treatment has been well demonstrated, its underlying mechanisms have still not been fully elucidated. The occurrence and development of AML have been extensively linked to the imbalance of epigenetic modification in numerous studies, yet further research is still required to elucidate how TCM exerts its regulatory role. Furthermore, the objective, scientific, and systematic evaluation of the effectiveness of cancer treatment remains a big challenge in clinical trials design for TCM, which hinders the international development of TCM for cancer treatment. The tumor response evaluation system in TCM is gradually consistent with WM, as both emphasize the integration of soft endpoints (such as quality of life and clinical benefits) and hard endpoints (such as tumor remission rate and progression time). However, the research on cancer treatment with TCM still should adhere to its own theoretical and clinical systems, minimizing passive imitation of WM, in order to establish a cancer treatment model with Chinese characteristics. Establishing chinese medicine characteristic tumor response evaluation system is the key to promote internationalization of chinese medicine oncology.

Combining TCM with current epigenetic drugs (e.g., azacitidine, decitabine) offers a promising multi-targeted approach, which could address the complexities and heterogeneity of AML. Both TCM compounds and epigenetic drugs aim to modify disease processes at the molecular level, and their combination could lead to synergistic therapeutic effects. Some TCM compounds might enhance the efficacy of epigenetic drugs by affecting complementary pathways. For example, certain Chinese herbs are believed to modulate DNA methylation or histone modifications, which could strengthen the effects of epigenetic drugs targeting these processes. Resistance to therapy is a significant challenge in AML treatment. Combining TCM with epigenetic drugs might reduce the likelihood of resistance developing, as the multi-targeted approach makes it harder for cancer cells to bypass therapeutic blockades. Maintenance therapy for AML has undergone a transition from conventional chemotherapy drugs to targeted therapy and immunotherapy, representing one of the efficacious strategies in managing relapse in AML. The successful clinical trial of oral azacitidine (QUAZAR) suggests that maintenance therapy with survival benefits holds significant potential. TCM has long been used to alleviate side effects and improve the body’s resilience, which may serves as maintenance drug in AML therapy to improved overall survival and quality of life for AML patients, as well as enhance the body’s natural ability to recognize and attack AML cells. Besides, by detecting changes in epigenetic markers in AML patients, the disease progression of patients can be more accurately assessed, providing a crucial foundation for formulating personalized treatment plans. Through analysis of a patient’s epigenetic lineage, appropriate therapeutic targets and drug combinations can be selected, ultimately enhancing treatment efficacy while minimizing adverse reactions. This comprehensive approach represents a promising new direction for addressing hematologic malignancies like AML.

## 5 Conclusion

Aberrant epigenetic regulation plays a pivotal role in the pathogenesis and progression of AML, particularly involving DNA methylation, histone acetylation, and dysregulated miRNA expression. Numerous TCM and their active constituents have demonstrated epigenetic modulatory effects in the treatment of AML. With the rapid advance on sequencing technology, the disease progression of AML patients will be more accurately assessed to formulate personalized therapy, ultimately enhancing treatment efficacy while minimizing adverse reactions. Additionally, the integration of TCM and modern epigenetic therapy represents a promising strategy for the treatment of AML, which is expected to yield significant clinical benefits.

## References

[B1] Alvarez M. C. Maso V. Torello C. O. Ferro K. P. Saad S. T. O. (2018). The polyphenol quercetin induces cell death in leukemia by targeting epigenetic regulators of pro-apoptotic genes. Clin. Epigenetics 10, 139. 10.1186/s13148-018-0563-3 30409182 PMC6225654

[B2] Barneda-Zahonero B. Parra M. (2012). Histone deacetylases and cancer. Mol. Oncol. 6, 579–589. 10.1016/j.molonc.2012.07.003 22963873 PMC5528343

[B3] Baylin S. B. Jones P. A. (2011). A decade of exploring the cancer epigenome - biological and translational implications. Nat. Rev. Cancer 11, 726–734. 10.1038/nrc3130 21941284 PMC3307543

[B4] Bernt K. M. Zhu N. Sinha A. U. Vempati S. Faber J. Krivtsov A. V. (2011). MLL-rearranged leukemia is dependent on aberrant H3K79 methylation by DOT1L. Cancer Cell 20, 66–78. 10.1016/j.ccr.2011.06.010 21741597 PMC3329803

[B5] Beyer M. Romanski A. Mustafa A. M. Pons M. Büchler I. Vogel A. (2019). HDAC3 activity is essential for human leukemic cell growth and the expression of β-catenin, MYC, and WT1. Cancers (Basel) 11, 1436. 10.3390/cancers11101436 31561534 PMC6826998

[B6] Borutinskaitė V. Virkšaitė A. Gudelytė G. Navakauskienė R. (2018). Green tea polyphenol EGCG causes anti-cancerous epigenetic modulations in acute promyelocytic leukemia cells. Leuk. Lymphoma 59, 469–478. 10.1080/10428194.2017.1339881 28641467

[B7] Bueso-Ramos C. E. Kanagal-Shamanna R. Routbort M. J. Hanson C. A. (2015). Therapy-related myeloid neoplasms. Am. J. Clin. Pathol. 144, 207–218. 10.1309/ajcpu1jo2lytwuav 26185306

[B8] Cao R. Wang L. Wang H. Xia L. Erdjument-Bromage H. Tempst P. (2002). Role of histone H3 lysine 27 methylation in Polycomb-group silencing. Science 298, 1039–1043. 10.1126/science.1076997 12351676

[B9] Chan S. M. Majeti R. (2013). Role of DNMT3A, TET2, and IDH1/2 mutations in pre-leukemic stem cells in acute myeloid leukemia. Int. J. Hematol. 98, 648–657. 10.1007/s12185-013-1407-8 23949914 PMC5542003

[B10] Chen Z. Zhuang X. (2024). Meta-analysis of the efficacy and safety of integrated Chinese and Western medicine in the treatment of acute myeloid leukaemia in elderly people. Heliyon 10, e23398. 10.1016/j.heliyon.2023.e23398 38226271 PMC10788428

[B11] Corces-Zimmerman M. R. Majeti R. (2014). Pre-leukemic evolution of hematopoietic stem cells: the importance of early mutations in leukemogenesis. Leukemia 28, 2276–2282. 10.1038/leu.2014.211 25005245 PMC4262622

[B12] Cragg G. M. Newman D. J. (2009). Nature: a vital source of leads for anticancer drug development. Phytochem. Rev. 8, 313–331. 10.1007/s11101-009-9123-y

[B13] Cui D. Xu X. (2018). DNA methyltransferases, DNA methylation, and age-associated cognitive function. Int. J. Mol. Sci. 19, 1315. 10.3390/ijms19051315 29710796 PMC5983821

[B14] Dahariya S. Paddibhatla I. Kumar S. Raghuwanshi S. Pallepati A. Gutti R. K. (2019). Long non-coding RNA: classification, biogenesis and functions in blood cells. Mol. Immunol. 112, 82–92. 10.1016/j.molimm.2019.04.011 31079005

[B15] Deveau A. P. Forrester A. M. Coombs A. J. Wagner G. S. Grabher C. Chute I. C. (2015). Epigenetic therapy restores normal hematopoiesis in a zebrafish model of NUP98-HOXA9-induced myeloid disease. Leukemia 29, 2086–2097. 10.1038/leu.2015.126 26017032

[B16] Di Croce L. Raker V. A. Corsaro M. Fazi F. Fanelli M. Faretta M. (2002). Methyltransferase recruitment and DNA hypermethylation of target promoters by an oncogenic transcription factor. Science 295, 1079–1082. 10.1126/science.1065173 11834837

[B17] DiNardo C. D. Ravandi F. Agresta S. Konopleva M. Takahashi K. Kadia T. (2015). Characteristics, clinical outcome, and prognostic significance of IDH mutations in AML. Am. J. Hematol. 90, 732–736. 10.1002/ajh.24072 26016821 PMC4612499

[B18] Döhner H. Estey E. Grimwade D. Amadori S. Appelbaum F. R. Büchner T. (2017). Diagnosis and management of AML in adults: 2017 ELN recommendations from an international expert panel. Blood 129, 424–447. 10.1182/blood-2016-08-733196 27895058 PMC5291965

[B19] Döhner H. Weisdorf D. J. Bloomfield C. D. (2015). Acute myeloid leukemia. N. Engl. J. Med. 373, 1136–1152. 10.1056/NEJMra1406184 26376137

[B20] Eberharter A. Becker P. B. (2002). Histone acetylation: a switch between repressive and permissive chromatin. Second in review series on chromatin dynamics. EMBO Rep. 3, 224–229. 10.1093/embo-reports/kvf053 11882541 PMC1084017

[B21] Egan G. Schimmer A. D. (2023). Contribution of metabolic abnormalities to acute myeloid leukemia pathogenesis. Trends Cell Biol. 33, 455–462. 10.1016/j.tcb.2022.11.004 36481232

[B22] Figueroa M. E. Abdel-Wahab O. Lu C. Ward P. S. Patel J. Shih A. (2010). Leukemic IDH1 and IDH2 mutations result in a hypermethylation phenotype, disrupt TET2 function, and impair hematopoietic differentiation. Cancer Cell 18, 553–567. 10.1016/j.ccr.2010.11.015 21130701 PMC4105845

[B23] Ghaffari S. H. Bashash D. Dizaji M. Z. Ghavamzadeh A. Alimoghaddam K. (2012). Alteration in miRNA gene expression pattern in acute promyelocytic leukemia cell induced by arsenic trioxide: a possible mechanism to explain arsenic multi-target action. Tumour Biol. 33, 157–172. 10.1007/s13277-011-0259-1 22072212

[B24] Hassan H. E. Carlson S. Abdallah I. Buttolph T. Glass K. C. Fandy T. E. (2015). Curcumin and dimethoxycurcumin induced epigenetic changes in leukemia cells. Pharm. Res. 32, 863–875. 10.1007/s11095-014-1502-4 25186441 PMC11173366

[B25] Hassan H. E. Keita J. A. Narayan L. Brady S. M. Frederick R. Carlson S. (2016). The combination of dimethoxycurcumin with DNA methylation inhibitor enhances gene re-expression of promoter-methylated genes and antagonizes their cytotoxic effect. Epigenetics 11, 740–749. 10.1080/15592294.2016.1226452 27588609 PMC5094623

[B26] Hassani S. Khaleghian A. Ahmadian S. Alizadeh S. Alimoghaddam K. Ghavamzadeh A. (2018). Redistribution of cell cycle by arsenic trioxide is associated with demethylation and expression changes of cell cycle related genes in acute promyelocytic leukemia cell line (NB4). Ann. Hematol. 97, 83–93. 10.1007/s00277-017-3163-y 29159499

[B27] Huang L. Li H. Xie D. Shi T. Wen C. (2017). Personalizing Chinese medicine by integrating molecular features of diseases and herb ingredient information: application to acute myeloid leukemia. ONCOTARGET 8, 43579–43591. 10.18632/oncotarget.16983 28454110 PMC5522171

[B28] Iravani Saadi M. Moayedi J. Hosseini F. Rostamipour H. A. Karimi Z. Rahimian Z. (2023). The effects of resveratrol, gallic acid, and piperine on the expression of miR-17, miR-92b, miR-181a, miR-222, BAX, BCL-2, MCL-1, WT1, c-kit, and CEBPA in human acute myeloid leukemia cells and their roles in apoptosis. Biochem. Genet. 10.1007/s10528-023-10582-8 38062274

[B29] Jadhav V. Ray P. Sachdeva G. Bhatt P. (2016). Biocompatible arsenic trioxide nanoparticles induce cell cycle arrest by p21(WAF1/CIP1) expression via epigenetic remodeling in LNCaP and PC3 cell lines. Life Sci. 148, 41–52. 10.1016/j.lfs.2016.02.042 26883975

[B30] Jaenisch R. (1997). DNA methylation and imprinting: why bother? Trends Genet. 13, 323–329. 10.1016/s0168-9525(97)01180-3 9260519

[B31] Jezek M. Green E. M. (2019). Histone modifications and the maintenance of telomere integrity. Cells 8, 199. 10.3390/cells8020199 30823596 PMC6407025

[B32] Khaleghian A. Ghaffari S. H. Ahmadian S. Alimoghaddam K. Ghavamzadeh A. (2014). Metabolism of arsenic trioxide in acute promyelocytic leukemia cells. J. Cell Biochem. 115, 1729–1739. 10.1002/jcb.24838 24819152

[B33] Khwaja A. Bjorkholm M. Gale R. E. Levine R. L. Jordan C. T. Ehninger G. (2016). Acute myeloid leukaemia. Nat. Rev. Dis. Prim. 2, 16010. 10.1038/nrdp.2016.10 27159408

[B34] Langdon K. Cosentino S. Wawryk O. (2024). Superiority of anthracycline-free treatment in standard-risk acute promyelocytic leukemia: a systematic review and comparative epidemiological analysis. Cancer Rep. Hob. 7, e2035. 10.1002/cnr2.2035 PMC10953833 38507294

[B35] Lee W. J. Chen Y. R. Tseng T. H. (2011). Quercetin induces FasL-related apoptosis, in part, through promotion of histone H3 acetylation in human leukemia HL-60 cells. Oncol. Rep. 25, 583–591. 10.3892/or.2010.1097 21165570

[B36] Lei Y. Li X. Zhu L. (2024). Matrine regulates miR-495-3p/miR-543/PDK1 axis to repress the progression of acute myeloid leukemia via the Wnt/β-catenin pathway. Chem. Biol. Drug Des. 103, e14441. 10.1111/cbdd.14441 38230785

[B37] Leisch M. Pfeilstöcker M. Stauder R. Heibl S. Sill H. Girschikofsky M. (2022). Adverse events in 1406 patients receiving 13,780 cycles of azacitidine within the Austrian registry of hypomethylating agents-A prospective cohort study of the AGMT study-group. Cancers (Basel) 14, 2459. 10.3390/cancers14102459 35626063 PMC9140081

[B38] Li C. Dong L. Su R. Bi Y. Qing Y. Deng X. (2020b). Homoharringtonine exhibits potent anti-tumor effect and modulates DNA epigenome in acute myeloid leukemia by targeting SP1/TET1/5hmC. Haematologica 105, 148–160. 10.3324/haematol.2018.208835 30975912 PMC6939512

[B39] Li H. Wang Y. Xu W. Dong L. Guo Y. Bi K. (2015). Arsenic trioxide inhibits DNA methyltransferase and restores TMS1 gene expression in K562 cells. Acta Haematol. 133, 18–25. 10.1159/000362683 24993472

[B40] Li J. Chen P. Sinogeeva N. Gorospe M. Wersto R. P. Chrest F. J. (2002). Arsenic trioxide promotes histone H3 phosphoacetylation at the chromatin of CASPASE-10 in acute promyelocytic leukemia cells. J. Biol. Chem. 277, 49504–49510. 10.1074/jbc.M207836200 12388546

[B41] Li K. K. Luo L. F. Shen Y. Xu J. Chen Z. Chen S. J. (2013). DNA methyltransferases in hematologic malignancies. Semin. Hematol. 50, 48–60. 10.1053/j.seminhematol.2013.01.005 23507483

[B42] Li M. L. Wang Y. Xu Y. N. Lu Q. Y. (2020a). Overexpression of LncRNA-HOTAIR promotes chemoresistance in acute leukemia cells. Int. J. Clin. Exp. Pathol. 13, 3044–3051. 33425105 PMC7791381

[B43] Liang H. Li X. Wang L. Yu S. Xu Z. Gu Y. (2013). MicroRNAs contribute to promyelocyte apoptosis in As2O3-treated APL cells. Cell Physiol. Biochem. 32, 1818–1829. 10.1159/000356615 24356076

[B44] Liao M. Dong Q. Chen R. Xu L. Jiang Y. Guo Z. (2021). Oridonin inhibits DNMT3A R882 mutation-driven clonal hematopoiesis and leukemia by inducing apoptosis and necroptosis. Cell Death Discov. 7, 297. 10.1038/s41420-021-00697-5 34663800 PMC8523644

[B45] Liu H. (2021). Emerging agents and regimens for AML. J. Hematol. Oncol. 14, 49. 10.1186/s13045-021-01062-w 33757574 PMC7989091

[B46] Liu J. M. Li M. Luo W. Sun H. B. (2021). Curcumin attenuates Adriamycin-resistance of acute myeloid leukemia by inhibiting the lncRNA HOTAIR/miR-20a-5p/WT1 axis. Lab. Invest. 101, 1308–1317. 10.1038/s41374-021-00640-3 34282279

[B47] Liu X. Jia X. Yuan H. Ma K. Chen Y. Jin Y. (2015a). DNA methyltransferase 1 functions through C/ebpa to maintain hematopoietic stem and progenitor cells in zebrafish. J. Hematol. Oncol. 8, 15. 10.1186/s13045-015-0115-7 25886310 PMC4372312

[B48] Liu Z. H. Li J. Xia J. Jiang R. Zuo G. W. Li X. P. (2015b). Ginsenoside 20(s)-Rh2 as potent natural histone deacetylase inhibitors suppressing the growth of human leukemia cells. Chem. Biol. Interact. 242, 227–234. 10.1016/j.cbi.2015.10.014 26482938

[B49] Luo H. Vong C. T. Chen H. Gao Y. Lyu P. Qiu L. (2019). Naturally occurring anti-cancer compounds: shining from Chinese herbal medicine. Chin. Med. 14, 48. 10.1186/s13020-019-0270-9 31719837 PMC6836491

[B50] Ma S. Huang Q. Hu Q. Gao R. Lan J. Yu X. (2022). Ginsenoside Rk3 inhibits the extramedullary infiltration of acute monocytic leukemia cell via miR-3677-5p/CXCL12 Axis. Evid. Based Complement. Altern. Med. 2022, 3065464. 10.1155/2022/3065464 PMC9788880 36569343

[B51] Ma X. Li C. Xu H. Wang K. Dong P. Zhou Y. (2021). Analysis on clinical medication rules in treatment of chronic myeloid leukemia based on data mining. Chin J Inf Tradit Chin Med. 28, 30–35.

[B52] Maimaitiyiming Y. Wang Q. Q. Hsu C. H. Naranmandura H. (2020). Arsenic induced epigenetic changes and relevance to treatment of acute promyelocytic leukemia and beyond. Toxicol. Appl. Pharmacol. 406, 115212. 10.1016/j.taap.2020.115212 32882258

[B53] Milne T. A. Briggs S. D. Brock H. W. Martin M. E. Gibbs D. Allis C. D. (2002). MLL targets SET domain methyltransferase activity to Hox gene promoters. Mol. Cell 10, 1107–1117. 10.1016/s1097-2765(02)00741-4 12453418

[B54] Mizuno S. Chijiwa T. Okamura T. Akashi K. Fukumaki Y. Niho Y. (2001). Expression of DNA methyltransferases DNMT1, 3A, and 3B in normal hematopoiesis and in acute and chronic myelogenous leukemia. Blood 97, 1172–1179. 10.1182/blood.v97.5.1172 11222358

[B55] Moradzadeh M. Roustazadeh A. Tabarraei A. Erfanian S. Sahebkar A. (2018). Epigallocatechin-3-gallate enhances differentiation of acute promyelocytic leukemia cells via inhibition of PML-RARα and HDAC1. Phytother. Res. 32, 471–479. 10.1002/ptr.5990 29193405

[B56] Müller B. M. Jana L. Kasajima A. Lehmann A. Prinzler J. Budczies J. (2013). Differential expression of histone deacetylases HDAC1, 2 and 3 in human breast cancer-overexpression of HDAC2 and HDAC3 is associated with clinicopathological indicators of disease progression. BMC Cancer 13, 215. 10.1186/1471-2407-13-215 23627572 PMC3646665

[B57] Nayak S. Shen M. Bunaciu R. P. Bloom S. E. Varner J. D. Yen A. (2010). Arsenic trioxide cooperates with all trans retinoic acid to enhance mitogen-activated protein kinase activation and differentiation in PML-RARalpha negative human myeloblastic leukemia cells. Leuk. Lymphoma 51, 1734–1747. 10.3109/10428194.2010.501535 20615082 PMC4896300

[B58] Nie Z. Y. Zhao M. H. Cheng B. Q. Pan R. F. Wang T. R. Qin Y. (2020). Tanshinone IIA regulates human AML cell proliferation, cell cycle, and apoptosis through miR-497-5p/AKT3 axis. Cancer Cell Int. 20, 379. 10.1186/s12935-020-01468-5 32782437 PMC7412841

[B59] Nuno K. A. Azizi A. Köhnke T. Lareau C. A. Ediwirickrema A. Ryan Corces M. (2023). Convergent epigenetic evolution drives relapse in acute myeloid leukemia. bioRxiv. 10.1101/2023.10.10.561642 PMC11034943 38647535

[B60] Ohgami R. S. Arber D. A. (2015). The diagnostic and clinical impact of genetics and epigenetics in acute myeloid leukemia. Int. J. Lab. Hematol. 37 (Suppl. 1), 122–132. 10.1111/ijlh.12367 25976970

[B61] Pan C. Zhu D. Zhuo J. Li L. Wang D. Zhang C. Y. (2016). Role of signal regulatory protein α in arsenic trioxide-induced promyelocytic leukemia cell apoptosis. Sci. Rep. 6, 23710. 10.1038/srep23710 27010069 PMC4806322

[B62] Papaemmanuil E. Gerstung M. Bullinger L. Gaidzik V. I. Paschka P. Roberts N. D. (2016). Genomic classification and prognosis in acute myeloid leukemia. N. Engl. J. Med. 374, 2209–2221. 10.1056/NEJMoa1516192 27276561 PMC4979995

[B63] Ramirez T. Brocher J. Stopper H. Hock R. (2008). Sodium arsenite modulates histone acetylation, histone deacetylase activity and HMGN protein dynamics in human cells. Chromosoma 117, 147–157. 10.1007/s00412-007-0133-5 17999076

[B64] Sato H. Wheat J. C. Steidl U. Ito K. (2016). DNMT3A and TET2 in the pre-leukemic phase of hematopoietic disorders. Front. Oncol. 6, 187. 10.3389/fonc.2016.00187 27597933 PMC4992944

[B65] Sawesi S. Malkaram S. A. Abd Elmageed Z. Y. Fandy T. E. (2022). Modulation of the activity of histone lysine methyltransferases and demethylases by curcumin analog in leukaemia cells. J. Cell Mol. Med. 26, 5624–5633. 10.1111/jcmm.17589 36300880 PMC9667515

[B66] Schaefer C. B. Ooi S. K. Bestor T. H. Bourc'his D. (2007). Epigenetic decisions in mammalian germ cells. Science 316, 398–399. 10.1126/science.1137544 17446388

[B67] Shi X. Gao H. Y. Yan W. He X. W. Yang W. (2018). Effects of EGCG on proliferation, cell cycle and DAPK1 gene methylation of acute promyelocytic leukemia NB4 cell line. Zhongguo Shi Yan Xue Ye Xue Za Zhi 26, 1288–1293. 10.7534/j.issn.1009-2137.2018.05.006 30295240

[B68] Stelmach P. Trumpp A. (2023). Leukemic stem cells and therapy resistance in acute myeloid leukemia. Haematologica 108, 353–366. 10.3324/haematol.2022.280800 36722405 PMC9890038

[B69] Suganuma T. Workman J. L. (2011). Signals and combinatorial functions of histone modifications. Annu. Rev. Biochem. 80, 473–499. 10.1146/annurev-biochem-061809-175347 21529160

[B70] Sulkowski P. L. Corso C. D. Robinson N. D. Scanlon S. E. Purshouse K. R. Bai H. (2017). 2-Hydroxyglutarate produced by neomorphic IDH mutations suppresses homologous recombination and induces PARP inhibitor sensitivity. Sci. Transl. Med. 9, eaal2463. 10.1126/scitranslmed.aal2463 28148839 PMC5435119

[B71] Sun Y. Chen B. R. Deshpande A. (2018). Epigenetic regulators in the development, maintenance, and therapeutic targeting of acute myeloid leukemia. Front. Oncol. 8, 41. 10.3389/fonc.2018.00041 29527516 PMC5829038

[B72] Vitkeviciene A. Baksiene S. Borutinskaite V. Navakauskiene R. (2018). Epigallocatechin-3-gallate and BIX-01294 have different impact on epigenetics and senescence modulation in acute and chronic myeloid leukemia cells. Eur. J. Pharmacol. 838, 32–40. 10.1016/j.ejphar.2018.09.005 30194939

[B73] Vogelauer M. Wu J. Suka N. Grunstein M. (2000). Global histone acetylation and deacetylation in yeast. Nature 408, 495–498. 10.1038/35044127 11100734

[B74] Wang K. Chen Q. Shao Y. Yin S. Liu C. Liu Y. (2021). Anticancer activities of TCM and their active components against tumor metastasis. Biomed. Pharmacother. 133, 111044. 10.1016/j.biopha.2020.111044 33378952

[B75] Wang Q. Y. Zhong H. Chen F. Y. Zhang M. Y. Cai J. Y. Zhong J. H. (2016a). A preliminary study on epigenetic regulation of Acanthopanax senticosus in leukemia cell lines. Exp. Hematol. 44, 466–473. 10.1016/j.exphem.2016.03.002 26992299

[B76] Wang Z. Liu Y. Xue Y. Hu H. Ye J. Li X. (2016b). Berberine acts as a putative epigenetic modulator by affecting the histone code. Toxicol Vitro 36, 10–17. 10.1016/j.tiv.2016.06.004 27311644

[B77] Wu M. Jiang M. Xue M. Li Q. Cheng B. Huang M. (2020). Epigallocatechin gallate induces CHD5 gene demethylation to promote acute myeloid leukemia cell apoptosis *in vitro* by regulating p19(Arf)-p53-p21(Cip1) signaling pathway. Nan Fang. Yi Ke Da Xue Xue Bao 40, 1230–1238. 10.12122/j.issn.1673-4254.2020.09.02 32990229 PMC7544577

[B78] Xu Q. Y. Yu L. (2020). Epigenetic therapies in acute myeloid leukemia: the role of hypomethylating agents, histone deacetylase inhibitors and the combination of hypomethylating agents with histone deacetylase inhibitors. Chin. Med. J. Engl. 133, 699–715. 10.1097/cm9.0000000000000685 32044818 PMC7190219

[B79] Xu W. Yang H. Liu Y. Yang Y. Wang P. Kim S. H. (2011). Oncometabolite 2-hydroxyglutarate is a competitive inhibitor of α-ketoglutarate-dependent dioxygenases. Cancer Cell 19, 17–30. 10.1016/j.ccr.2010.12.014 21251613 PMC3229304

[B80] Yen K. Travins J. Wang F. David M. D. Artin E. Straley K. (2017). AG-221, a first-in-class therapy targeting acute myeloid leukemia harboring oncogenic IDH2 mutations. Cancer Discov. 7 (5), 478–493. 10.1158/2159-8290.Cd-16-1034 28193778

[B81] Yu J. Peng Y. Wu L.-C. Xie Z. Deng Y. Hughes T. (2013). Curcumin down-regulates DNA methyltransferase 1 and plays an anti-leukemic role in acute myeloid leukemia. PLos One 8, e55934. 10.1371/journal.pone.0055934 23457487 PMC3572185

[B82] Yu S. Ge Z. Chen W. Han J. (2023). Pyrrolidine dithiocarbamate enhances the cytotoxic effect of arsenic trioxide on acute promyelocytic leukemia cells. Comb. Chem. High. Throughput Screen 26, 2067–2076. 10.2174/1386207326666230123155944 36694317

[B83] Zappasodi R. Merghoub T. Wolchok J. D. (2018). Emerging concepts for immune checkpoint blockade-based combination therapies. Cancer Cell 33, 581–598. 10.1016/j.ccell.2018.03.005 29634946 PMC5896787

[B84] Zeidner J. F. (2020). Differentiating the differentiation syndrome associated with IDH inhibitors in AML. Clin. Cancer Res. 26, 4174–4176. 10.1158/1078-0432.Ccr-20-1820 32554513

[B85] Zhang P. P. Zhang F. Zhu K. Zhu J. F. Yuan Y. Yang Y. L. (2022). Matrine exerted an anti-tumor effect on acute myeloid leukemia via the lncRNA LINC01116/miR-592-mediated JAK/STAT pathway inactivation. Neoplasma 69, 123–135. 10.4149/neo_210802N1083 34881627

[B86] Zhang S. Zhang Q. Shi G. Yin J. (2018). MiR-182-5p regulates BCL2L12 and BCL2 expression in acute myeloid leukemia as a potential therapeutic target. Biomed. Pharmacother. 97, 1189–1194. 10.1016/j.biopha.2017.11.002 29136958

[B87] Zhu Y. Ouyang Z. Du H. Wang M. Wang J. Sun H. (2022). New opportunities and challenges of natural products research: when target identification meets single-cell multiomics. Acta Pharm. Sin. B 12, 4011–4039. 10.1016/j.apsb.2022.08.022 36386472 PMC9643300

